# A visual summary of the EUROCARE-4 results: a UK perspective

**DOI:** 10.1038/sj.bjc.6605400

**Published:** 2009-12-03

**Authors:** H Møller, K M Linklater, D Robinson

**Affiliations:** 1 Kings College London, Thames Cancer Registry, 1st Floor Capital House, 42 Weston Street, London SE1 3QD, UK

**Keywords:** cancer survival estimates, cancer survival in the United Kingdom or Europe, EUROCARE

## Abstract

**Background:**

This paper provides a one-page visual summary of the previously published relative survival estimates for 42 types of cancers in 23 countries in Europe.

**Methods:**

The cancer patients in these analyses were 15 years or older at the time of their diagnosis in the period 1995–1999. Follow-up was to the end of 2003 and relative survival estimates were computed by the cohort method.

**Results:**

The analysis of 1-year survival had good discriminatory power and visibly separated a group of countries with consistently high survival estimates (Switzerland, France, Sweden, Belgium and Italy) and another group of countries with lower estimates (Poland, Czech Republic, Ireland, Denmark and United Kingdom–Northern Ireland). After the first year, there was less variation between the countries.

**Conclusion:**

To more fully understand the UK situation, a rational comparison would select countries with data-quality, prosperity and healthcare systems that are similar to the United Kingdom. In otherwise comparable populations, a pronounced difference in 1-year survival is most likely to be due to variation in a strong prognostic factor, which exerts its effect in the short term. A likely explanation for the short-term survival deficit in the United Kingdom compared with the Nordic countries is a less favourable stage distribution in the United Kingdom. However, the present superficial analysis does not exclude possible functions for other factors relating to the organisation and quality of cancer care services.

Detailed results from the fourth generation of the EUROCARE cancer survival study were published in 2009 (Sant *et al*, 2009), providing information on survival from 42 different cancers in 23 European countries, and a number of journal articles relating to specific types of cancer have been published in a special issue of the *European Journal of Cancer* (EUROCARE Working Group, 2009). Apart from the specific clinical and public health interest in prognosis and survival for particular types of cancer, the collaborative survival study as a whole may help elucidate Europe-wide patterns of cancer survival, and provide interpretations and explanations that would not necessarily appear from detailed analyses of the individual diseases. Previous reports from the study noted important variations between larger geographical regions in Europe, increases in cancer survival with calendar time and associations with national health expenditure (Berrino *et al*, 2007; Verdecchia *et al*, 2007).

Several years ago, Ronald Damhuis from the cancer registry in Rotterdam suggested (personal communication, 2002) that variations in cancer survival between European countries might be more important in the short term (i.e. in the first year) after the cancer diagnosis, rather than in the longer term as measured by the then conventional 5- or 10-year cumulative survival estimates. This idea was substantiated by a detailed analysis of colorectal cancer survival in North West Europe (Engholm *et al*, 2007), and the routine reporting of the EUROCARE-4 study now includes survival estimates for the first year of follow-up (1-year survival) as well as an estimate for survival in the subsequent 4-year period, the latter called the 5-year survival conditional on surviving the first year (here abbreviated ‘5∣1-year conditional survival’).

This paper provides a one-page visual summary of 1-year survival and 5∣1-year conditional survival for 42 types of cancers in 23 countries in Europe.

## Materials and methods

We extracted the 1-year and 5∣1-year conditional survival estimates from the main EUROCARE-4 summary article (Sant *et al*, 2009). Both sets of results are estimates of relative survival, expressing probabilities of survival that have been corrected for the general, all-cause mortality in each population. Likewise, the estimates are age standardised to facilitate comparisons between countries with different age distributions (Corazziari *et al*, 2004). The cancer patients in these analyses were 15 years or older at the time of their diagnosis in the period 1995–1999. Follow-up was to the end of 2003 and relative survival estimates were computed by the cohort method (Brenner and Hakulinen, 2009).

**Figure 1:**
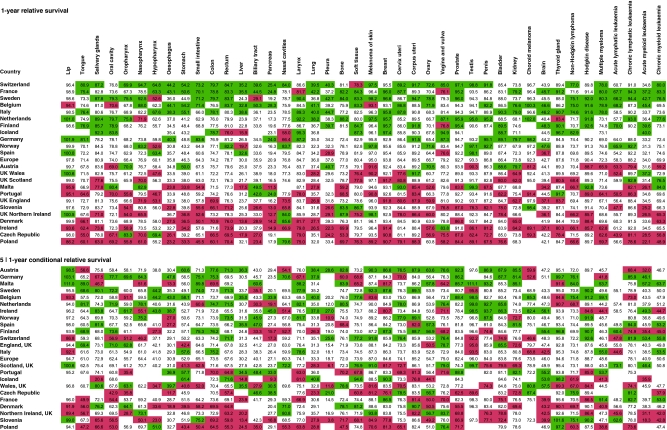
Summary analysis of relative survival for 42 types of cancer in 23 countries in Europe. The upper panel shows the 1-year survival estimates with the upper and lower quintiles within each cancer type indicated in green and red, respectively. The lower panel shows the corresponding analysis for 5-year relative survival, conditional on surviving the first year. For details, see text.

For each survival period, we identified the upper and lower quintiles of the distribution of survival estimates and colour coded the entries in Figure 1 accordingly. Green and red colours were used to identify the 20% of countries with the highest and the lowest, respectively, survival estimates for each type of cancer. We then sorted the countries in each of the two analyses by their scores of greens and reds across the cancer types. Not all the cancer-specific estimates were available for all the countries, and we constructed the analysis of the quintiles and the subsequent sorting of the countries, so as to be independent of the actual number of cancer types reported from each country.

Not all countries in Europe have complete coverage of cancer registration but, regardless of this, we summarised results under the heading of the name of each country. The EUROCARE-4 publication includes an analysis of the variation between cancer registries within countries (Sant *et al*, 2009). It is relevant to note that the United Kingdom and the Netherlands have little variability between their regional cancer registries. In France, Switzerland and Italy, where the population coverage of cancer registration is 11%, 27% and 25%, respectively, there is a higher degree of variability between their regional cancer registries. In consequence, the true survival statistics for the latter national populations are not well known at present, and the currently available estimates could change substantially if cancer registration was extended to the entire national populations.

## Results

Figure 1 summarises the results for the age-standardised 1-year relative survival (upper panel) and for the 5∣1-year conditional relative survival (lower panel). The analysis of 1-year survival had good discriminatory power, and visibly separated a group of countries with consistently high survival estimates (Switzerland, France, Sweden, Belgium and Italy) and another group of countries with lower estimates (Poland, Czech Republic, Ireland, Denmark and United Kingdom-Northern Ireland). The consistency of the pattern across cancer types was considerable, with only a few ‘outlying’ red cells in the upper part and only a few green cells in the lower part. Where such ‘exceptions’ occurred, they were most often in rare cancers, where the estimates are inherently less precise (e.g. soft tissue cancer in Switzerland and nasal cancer in Poland). The four UK countries were in the lower part of Figure 1.

The analysis of 5∣1-year conditional survival (i.e. cancer survival in the 4-year interval from 1 year after the cancer diagnosis to 5 years after diagnosis) was much less discriminatory (Figure 1; lower panel). The countries with the highest or the lowest overall rank also included some cancer types from the opposite end of the survival spectrum. For example, Austria had relatively low survival for cancer of the tongue and for four other cancer types, and Slovenia had relatively high survival from non-Hodgkin lymphoma and for five other types of cancer. The four UK countries were now higher and England was ranked above the European average.

## Discussion

The main question that this summary view of the data provokes concerns the origins of the consistent pattern observed in the short-term survival estimates.

An important feature of this analysis is that each type of cancer carries the same weight in the analysis, regardless of its level of incidence and its case fatality. One alternative analysis of the data would be to look at the aggregate survival from all types of cancer combined. However, such an analysis would be heavily influenced by the most common cancers (e.g. colorectal, lung, breast and prostate cancer) and it would miss details contributed by the less common cancers. The inclusion of very rare cancers with unstable survival estimates introduces random error into the current analysis, which could obscure a genuine pattern. On the other hand, their inclusion is not likely to spuriously generate any artificial pattern or structure in the data. Regardless, it may be useful to check any observation of high or low survival from the detailed analysis with an analysis of all cancers combined.

The 1-year and the 5∣1-year survival estimates are statistically independent: the value of one is not constrained by the value of the other. The two estimates represent short-term and medium-term mortality events, and they combine multiplicatively to give the conventional 5-year cumulative survival proportion. However, when it comes to interpretation, there is a sequential dependence between 1-year survival and 5∣1-year conditional survival. Surviving in the short term is a precondition for possibly enjoying the benefits of medium- and long-term survival and cure.

Models for understanding and describing any observed differences in cancer survival include at least three (not mutually exclusive) general mechanisms. Variation in cancer survival can be due to differences in the effectiveness of therapy and care for cancer patients (other things being equal); it can be due to differences in patient and disease characteristics, such as stage of disease or co-morbidity (other things being equal); and it can be independently brought about by systematic differences in the available data on cancer incidence and on deaths among cancer patients in the compared populations. It would be misguided to have an *a priori* preference for (or aversion to) any of these mechanisms.

### Countries with very high cancer survival

The analysis identifies a number of countries where cancer survival is consistently high in many types of cancer in 1-year survival, in 5∣1-year survival or in both: Switzerland, France, Sweden, Belgium, Italy, the Netherlands, Austria, Germany and Malta. Most of these countries have either partial coverage of cancer registration (ranging from 1% in Germany to 58% in Belgium) or small population size (Malta). Sweden may be seen as an example of the realisation of excellent survival outcomes through high-quality cancer care services and a cancer information and intelligence system that provides reliable cancer survival estimates. Sweden is a prosperous welfare state with publicly funded, well-organised and accessible health services. The population has a high level of education. Sweden has a system of central population registration and uses universal, unique person identifiers to resolve person-level identities and record linkages in its cancer registration and death certification processes. There is, however, one known feature of the Swedish cancer registration system that biases the survival estimates upwards. Cancer registration in Sweden does not use death certification as a means of primary case ascertainment, and the registry, therefore, misses a small proportion of fatal cancers that most other cancer registries would detect and register as ‘death-certificate-initiated’ cases (Parkin and Bray, 2009).

Another scenario where artificially high survival estimates occur is when the cancer registry has incomplete ascertainment of the deaths that occur among the persons on the register. In 2003, we did a similar visual summary analysis (unpublished; available from the authors) of the EUROCARE-3 data (Sant *et al*, 2003). Spain seemed to have very consistent, high cancer survival. In 2004, Carmen Martinez-Garcia from the cancer registry in Granada reported (oral presentation, Ragusa, September 2004) that the ascertainment of death events in some cancer registries in Spain had been inadequate. The erroneous data are included in the publication by Sant *et al* (2003) and no formal correction has been indexed in MEDLINE. The situation seems to have been corrected in EUROCARE-4 and Spain is no longer in the top bracket of cancer survival.

The example of Spain may apply elsewhere, however, because in many continental European countries, there are legislative and administrative barriers, which prevent cancer registries from gaining access to information about deaths. In this analysis, for example, the consistently high survival estimates in Belgium and the Netherlands may raise suspicion of incomplete ascertainment of deaths. In Belgium, in particular, there are surprisingly high 5-year survival estimates for a number of generally fatal types of cancer: oesophagus, pancreas, gallbladder and biliary tract, lung and acute myeloid leukaemia (Sant *et al*, 2009). Clinical excellence or a favourable stage distribution is certainly not ruled out in Belgium or elsewhere, but would be unlikely to apply simultaneously to all the most fatal types of cancer (Carpelan-Holmstrom *et al*, 2005).

Austria has very high survival estimates in the 5∣1-year analysis, but not in the 1-year estimates. In the 2003 analysis of EUROCARE-3 data, Austria had the highest survival estimates of all countries in Europe. The country was then represented by a single regional cancer registry comprising only 8% of the population, but in the current analysis, the new national cancer registry was used. How can a population plausibly have excellent cancer survival in the medium term, but average cancer survival in the short term? Clinical excellence or favourable stage distribution that acted in the medium term should act in the short term as well. One hypothesis, motivated by these data, may be that the completeness of ascertainment of deaths has changed with the introduction of the national cancer registration system.

France has a distinct pattern, which is the opposite of that in Austria: cancer survival in the short term is remarkably high, but after the first year survival changes to a very low level. Could it be that the French cancer registries are incomplete in the primary ascertainment of late stage cases, such as those cases that other registries may identify through the use of death certificates? The pattern is strange and not easy to attribute to a plausible clinical or behavioural scenario.

We do not wish to hypothesise about the cancer registration circumstances in each European country, but colleagues who are familiar with the details of cancer registration in Switzerland, Italy, Germany and other countries may perhaps have clues regarding the specific situations in these countries and the possible roles of cancer services, stage of disease and cancer registration processes in the survival estimates. There may be important lessons to learn from these countries, but we do not yet know what they are.

### Countries with very low cancer survival

Consistently low cancer survival is evident in the former communist east and central European countries: Poland, the Czech Republic and Slovenia. This is most likely due to a shortage of infrastructure and lack of financial resources for cancer care in these countries. The low survival from testicular cancer, for example, points to a shortage of platinum-based chemotherapeutic agents.

Denmark and Northern Ireland also seem to have low cancer survival in both periods of follow-up. The situation in Denmark with respect to cancer registration, ascertainment of deaths and access to tax-financed cancer care services is very similar to that in Sweden, yet the two countries are at opposite ends of the spectrum of cancer survival. The low cancer survival in Denmark has been confirmed in several detailed studies, but the reasons are not entirely clear (Engeland *et al*, 1998; Jensen *et al*, 2004; Mainz *et al*, 2009).

Northern Ireland is a small country and represents the lower end of the spectrum of estimates from the UK countries. Ireland also has consistent very low 1-year survival, but has very good 5∣1-year conditional survival. In the aggregate 5-year survival analysis, both Northern Ireland and Ireland have survival within the range of the other UK countries (Sant *et al*, 2009).

### UK perspective: who should we compare ourselves with?

It is well established that cancer survival in the United Kingdom is lower than we would expect from comparable European countries, but there is some contention about the most appropriate comparison. Clearly, there is the risk that some of the highest estimates are unrealistically high (e.g. Spain, Belgium, as discussed above) even if we are uncertain about the causes. For a comparison with the European average, see Thomson and Forman, 2009.

A rational comparison could select countries with data-quality, prosperity and healthcare system that are similar to the situation in the United Kingdom. The Nordic and the UK countries have many similar features: cancer registration covers the entire population in each country and is mostly of a high degree of completeness by international standards; the registries have access to death certification data and robust means of linking death information to the cancer registration record; the countries have a tax-financed healthcare system and cancer care services are free and accessible to the entire population. We may choose to omit Iceland from the comparison because of its very small population size, and we remain aware that the estimates from Sweden are too high because of the different policy regarding the use of death certificates in the cancer registration process.

There are two potentially strong biases that still might apply: the effect of the routine exclusion of death-certificate-only registrations from the survival analysis and the effect of the known incompleteness of at least some UK cancer registries (Bullard *et al*, 2000). A detailed analysis of data from the Thames Cancer Registry and the Finnish Cancer Registry showed that for a comparison of the UK and Nordic countries, the two effects act in opposite directions (Robinson *et al*, 2007).

Where, then, does this leave the assessment of cancer survival in the United Kingdom? Too low, unfortunately. In this analysis, all four UK countries have lower survival than Norway, Finland and Sweden, both in the 1-year and the 5∣1-year analysis. There is not much difference between the two analyses: in both, the UK countries are in the lower half and Norway, Sweden and Finland are in the upper half of Figure 1.

If we look at the alternative analysis of the survival from all cancers combined (Sant *et al*, 2009), a clear difference between the 1-year and the 5∣1-year conditional analysis appears. This analysis suggests that the UK deficit in survival compared with the Nordic countries is different in the two periods of follow-up. In the first year, the weighted 1-year survival is 63.8% in the United Kingdom and 74.6% in Norway, Sweden and Finland, a difference of 10.8 percentage points. In the 5∣1-year analysis, the difference is smaller: 72.1% in the United Kingdom *vs* 75.7% in the three Nordic countries, a difference of 3.6 percentage points.

In otherwise comparable populations, a pronounced difference in 1-year survival is most likely to be due to variation in a strong prognostic factor, which exerts its effect in the short term. A likely explanation for the short-term survival deficit in the United Kingdom compared with the Nordic countries is a less favourable stage distribution in the United Kingdom, but the present superficial analysis does not exclude possible functions for other factors relating to the organisation and quality of cancer care services.

## Conflict of interest

The authors declare no conflict of interest.
